# Curcumin inhibits proliferation, migration and neointimal formation of vascular smooth muscle via activating miR-22

**DOI:** 10.1080/13880209.2020.1781904

**Published:** 2020-07-06

**Authors:** Minghua Zhang, Yuntian Li, Hui Xie, Jing Chen, Shiming Liu

**Affiliations:** a The Second School of Clinical Medicine, Southern Medical University, Guangzhou, Guangdong, China; b Cardiovascular Department, The Fifth Affiliated Hospital, Guangzhou Medical University, Guangzhou, Guangdong, China; c Cardiovascular Center, 305 Hospital of Chinese People′s Liberation Army, Beijing, China; d Guangzhou Institute of Cardiovascular Disease, Guangdong Key Laboratory of Vascular Diseases, State Key Laboratory of Respiratory Disease, the Second Affiliated Hospital, Guangzhou Medical University, Guangzhou, Guangdong, China

**Keywords:** SP1, miR-22, curcumin, vascular injury

## Abstract

**Context:**

Curcumin has antitumor, antioxidative, anti-inflammatory, and anti-proliferative properties.

**Objective:**

To investigate the role of miR-22 during curcumin-induced changes in vascular smooth muscle cells (VSMC) and neointima formation in balloon-injured rat abdominal aorta.

**Materials and methods:**

Sprague-Dawley rats were randomised to the sham-operated (*n* = 10), operated control (injured, *n* = 10), and curcumin treatment (*n* = 10) groups. miR-22 expression was determined by real-time PCR. SP1 was assessed by western blot and real-time PCR. Rat aortic smooth muscle A7r5 cells were used to determine VSMC proliferation and migration, which were measured by the MTS, EdU staining, Transwell, and wound healing assays.

**Results:**

miR-22 levels declined following arterial balloon injury *in vivo* (48% at 3d, *p* < 0.05) and serum stimulation *in vitro* (45% at 24 h, *p* < 0.01). Functional studies revealed that miR-22 negatively regulated the proliferation and migration of VSMCs by directly targeting the SP1 transcription factor in VSMCs. Curcumin increased the expression of miR-22 (81%, *p* < 0.05) and decreased the protein expression of SP1 in VSMCs (25%, *p* < 0.05). miR-22 inhibition was found to attenuate the effects of curcumin on VSMC functions. Curcumin increased miR-22 (46%, *p* < 0.01), decreased the SP1 protein (19%, *p* < 0.05), and inhibited vascular neointimal area (48%, *p* < 0.01) *in vivo*.

**Discussion:**

The miR-22/SP1 pathway is involved in the protective role of curcumin during arterial balloon injury, but the mechanisms remain unclear.

**Conclusion:**

miR-22 is involved in the inhibitory effects of curcumin on VSMCs’ proliferation, migration and neointima hyperplasia after arterial balloon injury in rats. Curcumin could be used to prevent neointimal hyperplasia after angioplasty.

## Introduction

During interventional therapy for coronary heart disease, balloon dilatation, and stent implantation can damage the vascular endothelium by mechanical force, causing smooth muscle cells of the middle layer to migrate and proliferate, eventually leading to vascular stenosis or stent restenosis (Mintz et al. 1996; Curcio et al. 2011). The vascular smooth muscle cells (VSMCs) can be controlled during this process through various environmental stimuli (Alexander and Owens 2012). VSMC-specific genes become differentially expressed as the smooth muscle phenotype switches. For example, smooth muscle α-actin (α-SMA) and smooth muscle calponin levels decrease in the proliferative phenotype and increase in the contractile phenotype (Regan et al. 2000; Cheng et al. 2009).

Recent studies have demonstrated that the expression profiles of several miRNAs including miR-143/145 (Cordes et al. 2009), miR-133 (Torella et al. 2011), miR-23 (Iaconetti et al. 2015), and miR-221/222 (Davis et al. 2009) are dysregulated during the proliferation and differentiation of VSMCs. The specific molecular mechanisms that govern VSMC proliferation and re-endothelialization after stenting are not completely understood.

miRNAs are a group of short non-coding RNA that have critical functions in a range of human diseases, including diabetes, cardiovascular disease, kidney disease, and cancer (Paul et al. 2018). miR-22 was the first discovered miRNA with antitumor properties (Pandey and Picard 2009). Its expression has been subsequently identified to be dysregulated in multiple types of cancer, including gastric, breast, and liver cancer (Xiong et al. 2010; Kong et al. 2014). Recent studies have also revealed that miR-22 is richly expressed in the heart (Hu et al. 2012), where it plays a role in vascular remodelling (Zheng and Xu 2014), cardiac hypertrophy (Gurha et al. 2012; Huang et al. 2013), and spontaneous hypertension (Friese et al. 2013). It remains unclear as to whether miR-22 regulates the VSMC phenotype and neointimal formation in injured vascular.

Curcumin is an active compound derived from turmeric (*Curcuma longa* Linn. Zingiberaceae) (Hewlings and Kalman 2017). Curcumin has been shown to display a variety of activities, including antioxidant, anti-inflammatory, antiproliferation, and anti-angiogenic properties (Maheshwari et al. 2006; Anand et al. 2007). Recent studies have unveiled that curcumin can inhibit balloon injury-induced VSMC migration and neointimal formation (Yang et al. 2006; Yu and Lin 2010; Sheu et al. 2013). Curcumin also prevents the development of hypertension in an AngII-induced hypertensive model (Yao et al. 2016). In addition, curcumin has been shown to modulate the expression profiles of many miRNAs including miR-15a, miR-22, miR-125, miR-146, miR-200, and miR-49, and has been proposed as a therapeutic compound that can improve cancer treatment and other therapies (Shishodia 2013; Teiten et al. 2013; Li et al. 2018).

This study aimed to better understand the role of curcumin in modulating VSMC proliferation and migration. Mechanistically, we show that miR-22 inhibits VSMC proliferation and migration *in vitro* by targeting the transcriptional factor SP1. We established, both *in vitro* and *in vivo,* that miR-22/SP1 mediates the inhibitory effects of curcumin on VSMC proliferation and migration.

## Material and methods

### Drugs and reagents

Dulbecco’s modified Eagle medium was purchased from Invitrogen (CA, USA). Curcumin was obtained from Sigma (c7727, St. Louis, MO, USA); foetal bovine serum (FBS) was purchased from Gibco (CA, USA). Overexpression or inhibition of miR-22 was performed through the transfection of miR-22 mimics or miR-22 inhibitors from RiboBio (Guangzhou, China), and X-treme GENE siRNA was purchased from Roche (Basel, Switzerland). Cell proliferation assays were performed using the EdU Kit obtained from RiboBio (Guangzhou, China) and the MTS Kit purchased from Promega (WI, USA). Transwell chambers (8 μm) were purchased from Corning (NY, USA).

### Animals and artery balloon injury models

All operations strictly adhered to the guidance of the Institutional Animal Care and Use Committee. Adult male Sprague-Dawley rats (290–330 g) were purchased from the animal laboratory of the Health Science Centre of Southern Medical University. Thirty rats were randomised to the sham-operated group (sham, *n* = 10), operated but untreated control group (injured, *n* = 10), and curcumin treatment group (curcumin, *n* = 10). Balloon injury of the abdominal aorta was induced through the insertion of the deflated balloon through the left carotid artery down to the level of renal arteries. The rats were anaesthetised with 10% chloralhydrate (400 mg/kg, i.p.). The left carotid artery was catheterised with a 2-F deflated arterial balloon catheter that was placed distal to the renal arteries. Saline was injected into the tip of the anterior catheter to inflate the balloon, and the catheter was slowly pulled back to the diaphragm level. This procedure was repeated three times to ensure maximum removal of the vascular intima (Wang et al. 2004). The rats in the curcumin group were given 50 mg/kg of curcumin (2.5 mg of curcumin was dissolved in 10 mL of DMSO and mixed with 190 mL of olive oil) by intraperitoneal injection once a day for 14 days. The rats in the sham and injured groups received intraperitoneal injections of the solvent. On days 3 or 14, the abdominal aorta was removed from sacrificed rats and placed in 4% paraformaldehyde (PFA) tubes for histological analysis or snap-frozen for RNA and protein extraction.

### Cell culture

Rat aortic smooth muscle cells (A7r5) were purchased from the Shanghai Cell Bank (China). DMEM containing 10% FBS, 100 U/mL penicillin, and 100 µg/mL streptomycin were used to culture cells in a humidified environment in the presence of 5% CO_2_. The cells were plated at a density of 60%, and cultured for 12 hin serum-free DMEM. The cells were transfected with miR-22 mimics and/or miR-22 inhibitors for 6 in serum-free DMEM, following which fresh DMEM containing 10% FBS was added. All the experiments were repeated three times independently.

### VSMC proliferation assays

Proliferation assays were performed following the manufacturer’s protocols. For the EdU experiments, VSMC proliferation rates were calculated as the ratio of red-stained proliferating cells over the total cell number, assessed by fluorescence microscopy. For MTS assays, automatic measurements of the absorbance of cell samples were achieved by setting a threshold value of 490 nm (OD490) on a microplate reader. Absorbance values representative of cell proliferative capacity were compared between different treatment groups.

### VSMC migration assays

Transwell invasion and scratch wound healing assays were used to determine cell migration. For scratch wound healing assays, transfected VSMCs were grown to 100% confluency in 6-well plates and serum-starved for 12 h. We created two straight-line scratches using a 200 μL pipette at the centre of the 6-well plate, which was washed with PBS to remove floating cells and cell debris. Cells were stimulated in DMEM plus serum for 24 h. The scratch boundaries were imaged under an inverted microscope and analysed using the Image J software. For the transwell migration assays, transfected VSMCs (5 × 10^4^) were added to the upper chamber without FBS, and a medium containing 10% FBS was added to the lower chamber. Non-invasive cells were gently removed after 24 h. Cells invading the lower surface were fixed with 4% PFA, stained with gentian violet, and imaged on an inverted microscope. Cells that passed through the mesh were fixed with 4% PFA, and the invading cells were counted by microscopy following gentian violet staining.

### Dual-luciferase reporter assays

Cells were seeded on 96-well plate; 24 h later, they were transfected with wild-type and mutated SP1 3′-UTR under the control of the pRL-CMV control vector (Promega, USA) and miR-22 mimics or miR-22 negative controls. Then, Firefly plasmid (0.2 µg), renilla plasmid (0.004 µg), and transfection reagent (0.25 µL) were mixed and incubated at room temperature for 5 min. microRNA and transfection reagents were mixed so that the final concentration of microRNA was 100 nM incubated at room temperature for 5 min. The transfection mix and microRNA mix were added to the cells. The cells were changed with fresh culture medium 6 h later. After 48 h, PBS-washed cells were lysed using a passive lysis buffer. The supernatants were mixed with LR II and read for luminescence values. The Stop & Glo reagent was added, and the cells were read for luminescence values. Dual-luciferase reporter assays (Promega, USA) were used to evaluate the luciferase activity of the harvested cells.

### RNA isolation and real-time PCR

For RNA extraction, the cells were lysed in Trizol reagent (Invitrogen, USA) and the SYBR Green Real-time PCR (Takara, China) assay was performed to detect mature miR-22 or SP1 mRNA expression. SYBR green Real-time PCR was performed using the following primers: Calponin: sense, 5′-ACCAAGCGGCAGATCTTTGA-3′, antisense, 5′-CATCTGCAAGCTGACGTTGA-3′; α-SMA:sense, 5′-CTGCCTTGGTGTGTGACAATGG-3′, antisense, 5′-CGGGTACTTCAGGGTCAGGATTC-3′; SP1:sense, 5′-ACCTGGCGGTGATGGAAT-3′, antisense, 5′-GGTGGGTCTTGATATGCTTTG-3′; GAPDH:sense, 5′-GCACCGTCAAGGCTGAGAAC-3′, antisense, 5′-TGGTGAAGACGCCAGTGGA-3′; U6:sense, 5′-CTCGCTTCGGCAGCACA-3′, antisense, 5′-AACGCTTCACGAATTTGCGT-3′. U6 or GAPDH were used as endogenous controls. The 2^–ΔΔCT^ method was used for data processing. miR-22 mimic and miR-22 inhibitor were synthesised by Guangzhou RiboBio, and the sequences are as follows: MiR-22mimics sense: AAGCUGCCAGUUGAAGAACUGU; mimics antisense: AGUUCUUCAACUGGCAGCUUUU; inhibitor: ACAGUUCUUCAACUGGCAGCUU. The mimic NC and inhibitor NC sequences are proprietary (Guangzhou RiboBio).

### Western blot analysis

Cells were lysed in RIPA buffer (containing 0.1% PMSF), and the protein concentration of the cell lysates was assessed using BCA assays. Proteins were separated on SDS-PAGE gels and transferred to PVDF membranes. Membranes were blocked in 5% non-fat milk in TBST and probed overnight with mouse polyclonal SP1 or GAPDH primary antibodies (Santa Cruz, 1:1000). Membranes were labelled with HRP-conjugated secondary antibodies (1:5000) for 2 hat room temperature and visualised using the enhanced chemiluminescence reaction ECL Kit (Beyotime, China).

### Morphometric analysis for neointimal formation

Abdominal aortas were fixed in 4% PFA and subjected to paraffin embedding and sectioning for H&E staining. Sections (5-μm thick) were obtained at 100-μm intervals and mounted onto glass slides. Slides were H&E-stained to visualise vascular sections. Lumen, media, and neointimal were measured via computational image analysis (Image-Pro Plus).

### EdU staining

Cells were cultured in the EdU solution for 2 h. Cells were treated by Immunol Staining Fix Solution and Enhanced Immunostaining Permeabilization Buffer (RiboBio). Cells were then stained by Apollo and Hoechst33342.

### TUNEL staining

Cells were treated by Immunol Staining Fix Solution and Enhanced Immunostaining Permeabilization Buffer (Beyotime) and then treated with TUNEL detection buffer. Cells were analysed by flow cytometry or microscopy.

### Statistical analysis

All analyses were based on three independent experiments. Unpaired *t*-tests and one-way ANOVA tests with the LSD *post hoc* test were used to evaluate statistical significance. A *p*-value < 0.05 was deemed statistically significant.

## Results

### miR-22 expression during VSMC proliferation and in injured vascular walls

**Figure 1.**
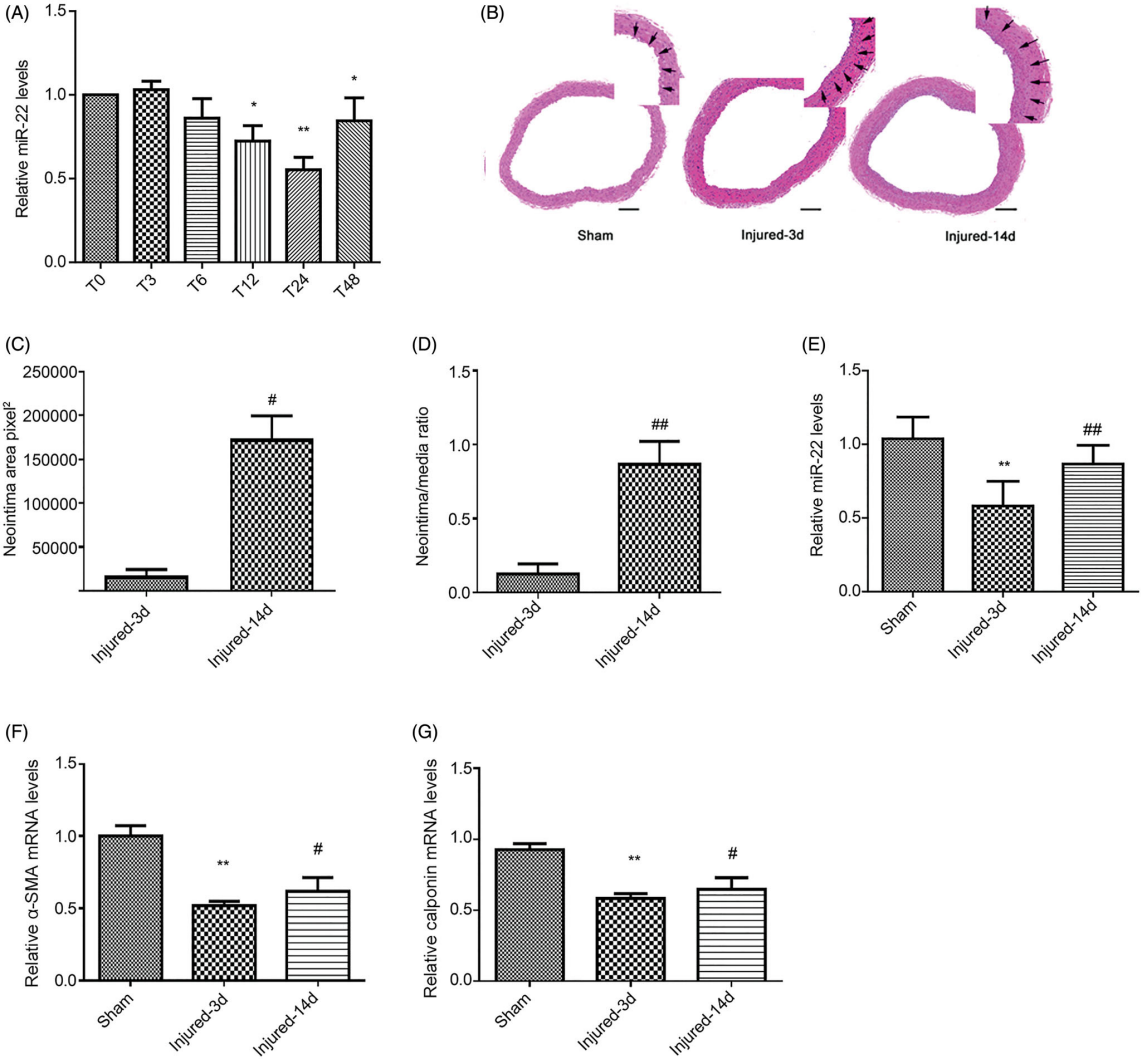
MiR-22 expression in VSMCs and rat balloon injury model. (A) miR-22 levels at 24 h of starvation (T0), 3, 6, 12, 24, and 48 h (T3, T6, T12, T24, and T48) during active proliferation following 10% FBS stimulation. (B) Arteries were stained with H&E 14 days post-balloon injury *in vivo* (scale bar = 100 μm) (magnification of the original microscope image: 100×; the enlarged portion was enlarged digitally from the original high-resolution microscope image). (C) Intima transection area after the arteries were balloon-injured. (D) The neointimal/media (N/M) ratio after arteries were balloon-injured. (E) mRNA level of miR-22 in the sham group, day 3 and day 14 after the injury. (F-G) mRNA level of α-SMA (F) and calponin (G) in the sham group, day 3 and day 14 after the injury. *p < 0.05; **p < 0.01: vs. T0 or Sham group; ^#^*p <* 0.05; ^##^*p <* 0.01: vs. injured-3d group. All values are expressed as the mean ± SD, *n* = 3 (A), *n* = 5 (B-G).

VSMCs were first starved with serum-free medium for 24 h and then treated with serum. miR-22 levels were detected by real-time PCR in rat serum-stimulated VSMCs (A7R5). We observed significant downregulation of miR-22 at 12 and 24 h after serum treatment (Figure 1(A)). At 48 h after serum stimulation, there was also a significant inhibition of miR-22 level in the cells, although it was much less than the inhibition that was observed at 12 and 24 h of stimulation. We next established the rat balloon injury model and observed increased neointimal area and neointimal/media ratio at 14 days after injury (Figure 1(B–D)). Furthermore, we assessed the levels of miR-22 in the rat balloon injury model and observed a decreased expression of miR-22 in the injury group at 3 days compared to the uninjured group, but increased expression of miR-22 at day 14 compared with day 3 (Figure 1(E)). α-SMA and calponin are well-established markers of VSMC differentiation. The expression of the two markers decreases as VSMCs proliferate. Interestingly, we observed a reduced expression of α-SMA and calponin at day 3 post the injury but increased expression at day 14 compared with day 3 (Figure 1(F–G)). Taken together, these data suggest that miR-22 expression is modulated during VSMC proliferation *in vitro* and *in vivo.*

### miR-22 inhibits VSMC proliferation and migration

We then transfected VSMCs with the miR-22 mimic or used the miR-22 inhibitor to test whether miR-22 negatively regulates the ability of VSMCs to proliferate and migrate. The concentration–response analysis showed that 25 nmol/L miR-22 mimic was appropriate for 10% FBS stimulation (Figure S1A), while 50 nmol/L miR-22 inhibitor was the appropriate concentration following 2% FBS stimulation compared with 10% FBS (Figure S1B–C). As 10% FBS stimulation leads to decreased expression of miR-22 in VSMCs, exogenous inhibitors failed to induce a further decrease. Thus, 2% FBS was used thereafter.

**Figure 2.**
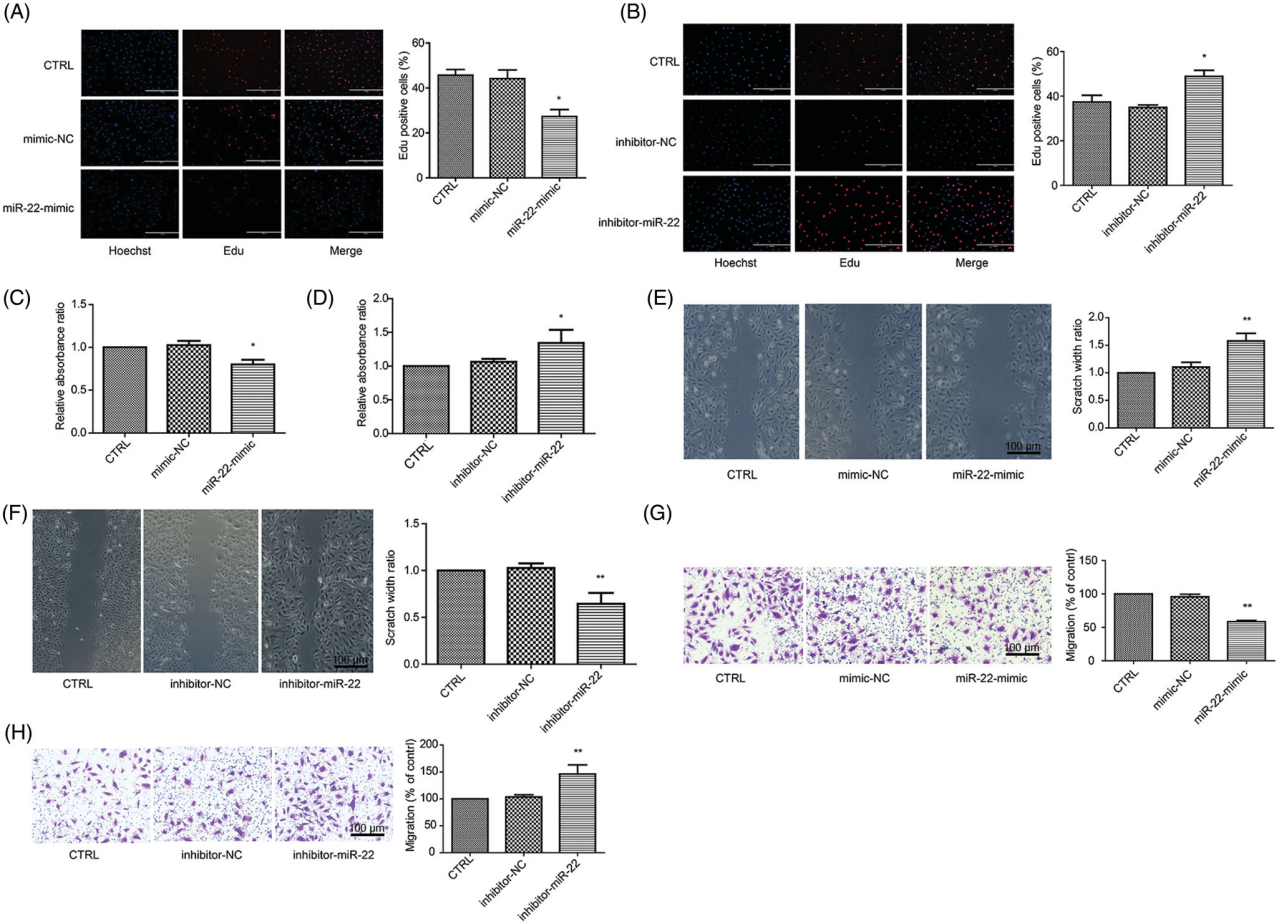
Role of miR-22 in VSMC proliferation and migration. (A) Representative images of Hochest and Edu staining and percentage of Edu-positive staining in control VSMCs, mimic NC-treated cells, and miR-22 mimic-treated cells. (B) Representative images of Hochest and Edu staining and percentage of Edu positive staining in control VSMCs, inhibitor NC-treated cells, and miR-22 inhibitor-treated cells. (C) Relative absorbance ratio by MTS assay in control VSMCs, mimic NC-treated cells, and miR-22 mimic-treated cells. (D) Relative absorbance ratio by MTS assay in control VSMCs, inhibitor NC-treated cells, and miR-22 inhibitor-treated cells. Representative images and quantification of the wound healing assay (E) and transwell migration assay (G) in control VSMCs, mimic NC-treated cells, and miR-22 mimic-treated cells. Representative images and quantification of the wound healing assay (F) and transwell migration assay (H) in control VSMCs, inhibitor NC-treated cells, and miR-22 inhibitor-treated cells. *p < 0.05; **p < 0.01: vs. CTRL group; All values are expressed as the mean ± SD, *n* = 3 (scale bar = 100 μm).

Interestingly, miR-22 mimic caused a significant decrease in the activity of VSMCs and reduced cell proliferation, as confirmed by MTS and EdU assays (Figure 2(A,C)). In contrast, miR-22 inhibition increased VSMC proliferation (Figure 2(B,D)). As expected, no significant differences were observed in VSMCs using mimic negative control (NC), inhibitor NC or NC transfection (Figure 2(A–D)). To assess the involvement of miR-22 during VSMC migration, wound healing (Figure 2(E–F)) and Transwell migration assays (Figure 2(G–H)) were performed. Consistent with the proliferation data, treatment of VSMCs with the miR-22 mimic reduced the cell migratory capacity in both assays, while treatment with the miR-22 inhibitor increased the migration (Figure 2(E–H)). No significant changes with the mimic NC or inhibitor NC in VSMCs were observed (Figure 2(E–H)).

### miR-22 negatively regulates SP1 expression by targeting its 3′-UTR

**Figure 3.**
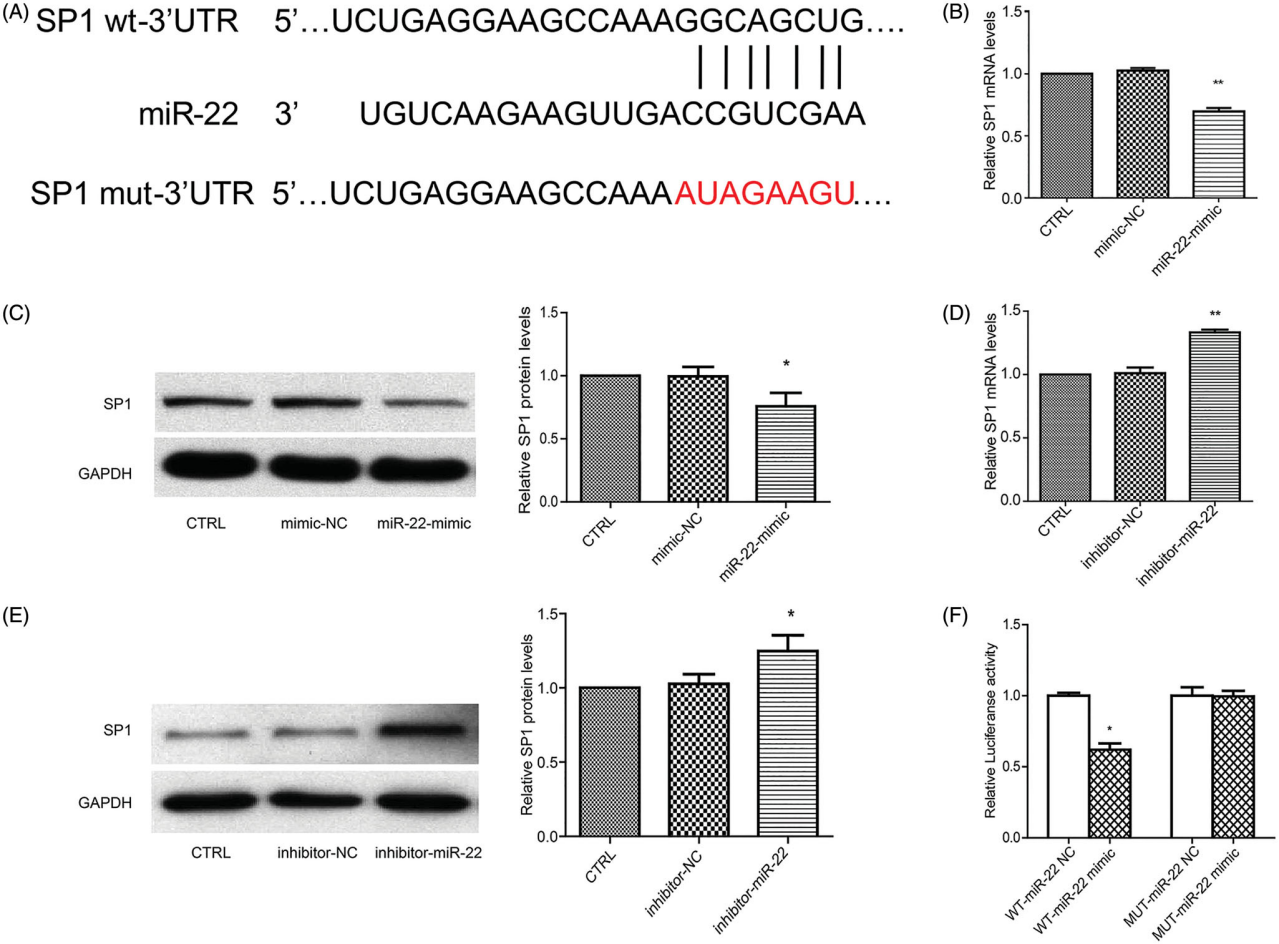
MiR-22 downregulates the expression of SP1. (A) Potential miR-22 binding sequence of SP1 3′-UTR and mutation sites. mRNA (B) and protein level (C) of SP1 in control VSMCs, mimic NC-treated cells, and miR-22 mimic-treated cells. mRNA (D) and protein level (E) of SP1 in control VSMCs, inhibitor NC-treated cells, and miR-22 inhibitor-treated cells. (F) Luciferase activity of wild-type and mutant SP1 3′-UTR reporters were determined in HEK293T cells. *p < 0.05; **p < 0.01: vs. CTRL or WT-miR-22 NC group; All values are expressed as the mean ± SD, *n* = 3.

Using the Pictar and TargetScan software from microRNA.org, we found that SP1 contains one putative miR-22 target site (Figure 3(A)). We, therefore, investigated the association between miR-22 and SP1 expression. miR-22 overexpression reduced SP1 expression under both mRNA and protein levels in VSMCs compared with the control cells (Figure 3(B–C)). By contrast, miR-22 inhibitor stimulation increased the mRNA and protein levels of SP1 (Figure 3(D–E)), confirming that miR-22 negatively regulates the expression of SP1. We confirmed these findings by transfecting miR-22 in HEK 293 T cells and showed that the luciferase activity of the wild-type SP1 3′-UTR was inhibited by the transfection (Figure 3(F)), the inhibition of which was rescued when the SP1 3′-UTR binding sites were mutated (Figure 3(F)). Taken together, these data demonstrate that miR-22 directly targets the SP1 3′-UTR and that SP1 is negatively regulated by miR-22 in VSMCs.

### Curcumin inhibits the proliferation and migration of vascular smooth muscle by activating miR-22

**Figure 4.**
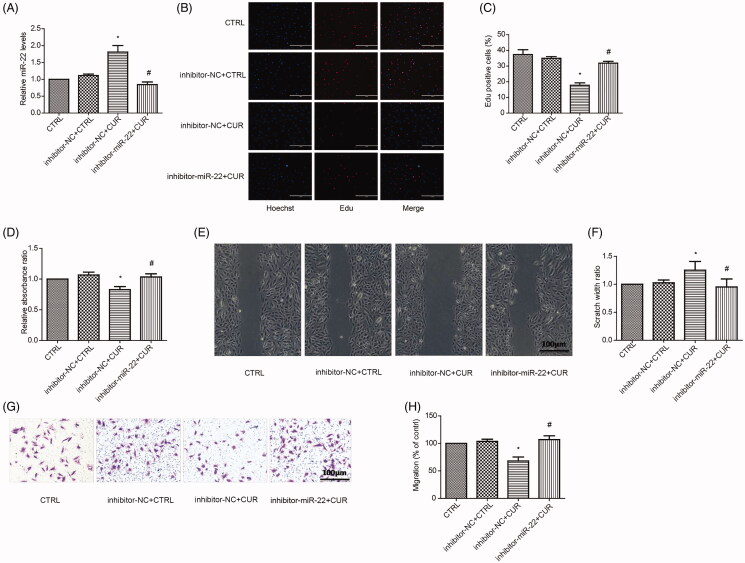
Curcumin inhibits the proliferation and migration of VSMCs via miR-22. (A) mRNA expression of miR-22 in control cells, inhibitor-NC + CTRL, inhibitor-NC + CUR or inhibitor-miR-22 + CUR-treated cells. Representative images of Hochest and Edu staining (B) and quantification of Edu-positive staining (C), relative absorbance ratio by MTS assay (D), representative images (E) and quantification (F) of scratch assay and representative images (G) and quantification (H) of transwell migration assay in control cells, inhibitor-NC + CTRL, inhibitor-NC + CUR or inhibitor-miR-22 + CUR-treated cells. *p < 0.05: vs. inhibitor-NC + CTRL; ^#^*p <* 0.05: vs. inhibitor-NC + CUR group. All values are expressed as the mean ± SD, *n* = 3 (scale bar =100 μm).

We next examined the effects of curcumin on miR-22 in VSMCs. A concentration-response analysis showed that the best curcumin dose chosen for the upregulated expression of miR-22 was 10 μM (Figure S2A–B). We showed that curcumin increased miR-22 expression (Figure 4(A)), which could be counteracted by the miR-22 inhibitor (Figure 4(A)). As shown by MTS and EdU assays, curcumin led to a drastic reduction in VSMC proliferation, which could also be reversed by the miR-22 inhibitor (Figure 4(B)). To further assess the effects of curcumin during VSMC migration, we performed wound healing (Figure 4(E,F)) and transwell migration assays (Figure 4(G,H)). Consistent with the proliferation data, curcumin reduced the migratory capacity of VSMCs compared with untreated control cells, while the miR-22 inhibitor partially rescued the inhibition (Figure 4(E,H)). These data show that curcumin inhibits VSMC proliferation and migration by miR-22.

### Curcumin increases α-SMA and calponin mRNA and decreases SP1 levels in VSMCs

**Figure 5.**
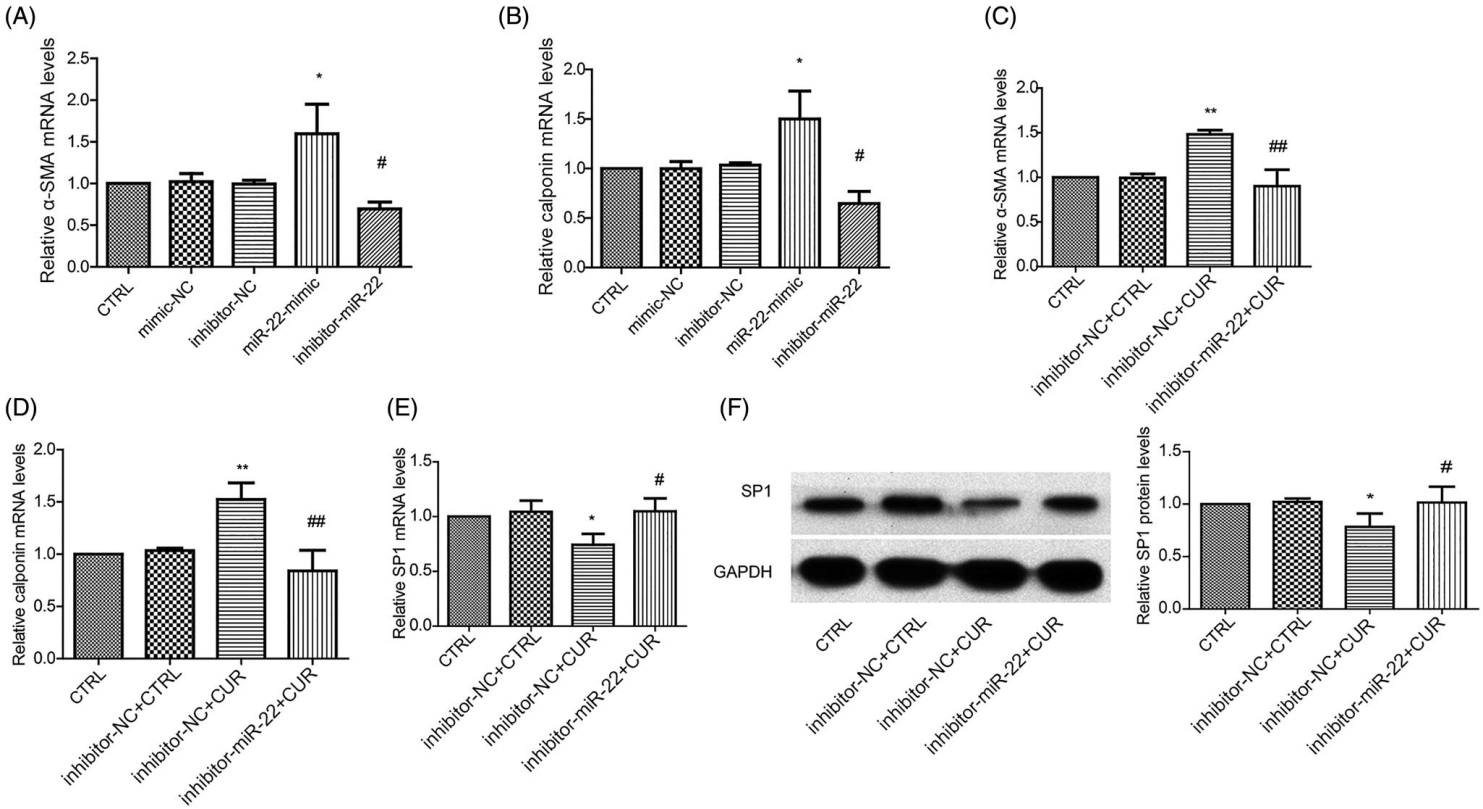
Curcumin increases α-SMA and calponin and decreases SP1 expression. mRNA expression of α-SMA (A) and calponin (B) in control cells, mimic-NC, inhibitor-NC, miR-22 mimic, miR-22 inhibitor-treated cells. mRNA expression of α-SMA (C), calponin (D), and mRNA (E) and protein (F) level of SP1 in control cells, inhibitor-NC + CTRL, inhibitor-NC + CUR and inhibitor-miR-22 + CUR-treated cells. *p < 0.05; **p < 0.01: vs. CTRL group or inhibitor-NC + CTRL group; ^#^*p <* 0.05; ^##^*p <* 0.01: vs. CTRL group or inhibitor-NC + CUR group. All values are expressed as the mean ± SD, *n* = 3.

We then measured the expression of α-SMA and calponin in the VSMCs. Real-time PCR results revealed that miR-22 mimic treatment upregulated α-SMA and calponin mRNA, while their expression was downregulated by miR-22 inhibition (Figure 5(A,B)). Furthermore, α-SMA and calponin mRNA levels in VSMCs were significantly increased following curcumin treatment, which was rescued by the miR-22 inhibitor (Figure 5(C,D)). To further clarify the effects of curcumin on smooth muscle cells, we assessed the impact of curcumin on SP1 expression. Curcumin treatment decreased mRNA and protein expression of SP1 (Figure 5(E,F)), an effect that was partially reversed by miR-22 inhibitors (Figure 5(G–I)). Taken together, these data reveal that in VSMCs, curcumin increases α-SMA and calponin mRNA expression and decrease SP1 levels in a miR-22-dependent manner.

### Curcumin suppresses balloon injury-induced neointima formation

**Figure 6.**
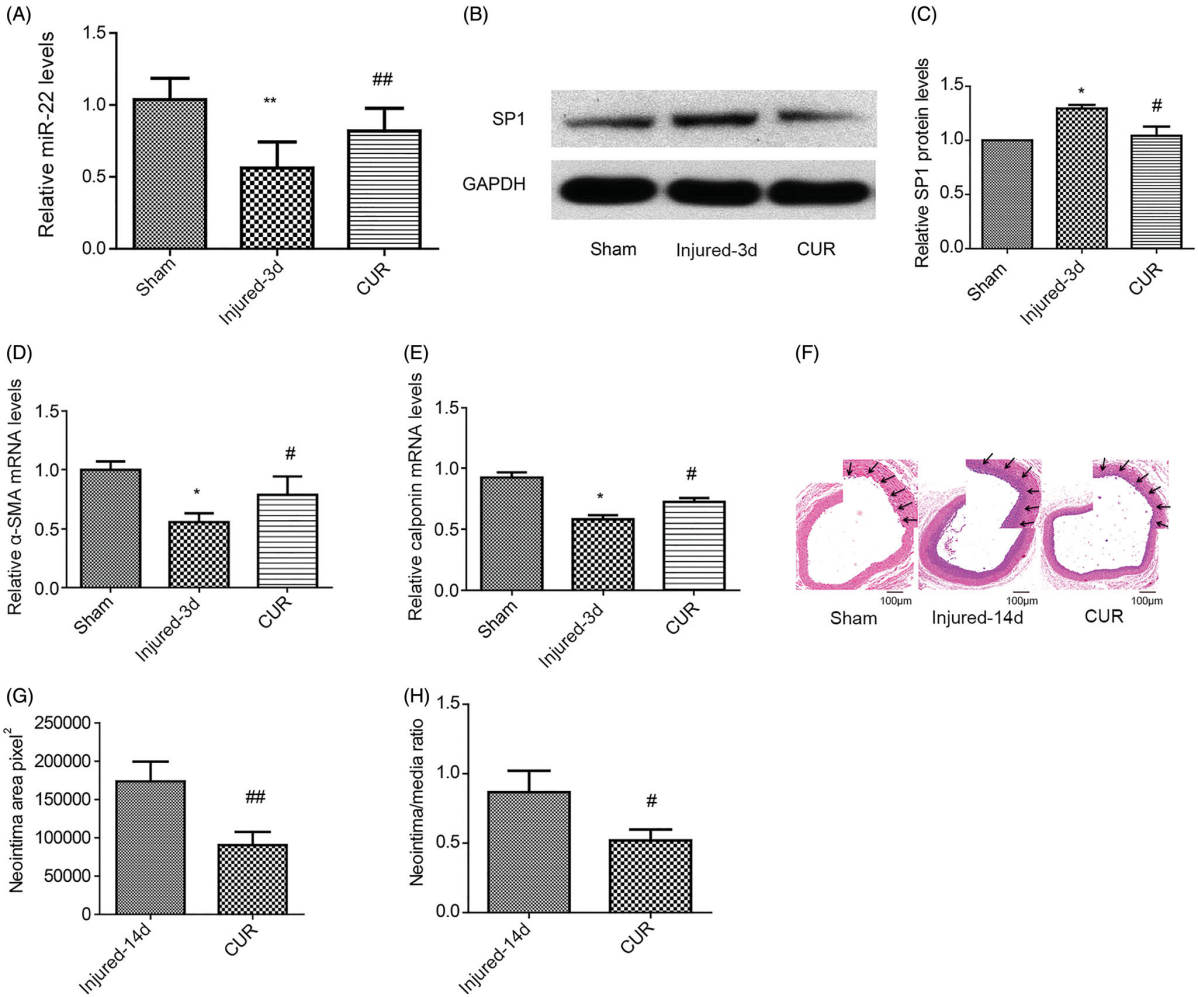
Protective role of curcumin in balloon injury models *in vivo.* mRNA expression of miR-22 (A) and protein levels (B-C) of SP1 in sham, injured, and curcumin-treated injured group 3 days post-balloon injury *in vivo*. mRNA expression of α-SMA (D) and calponin (E) in sham, injured, and curcumin-treated injured group 3 days post-balloon injury *in vivo*. (F) Representative images of H&E staining from sham, injured, and curcumin-treated injured group 14 days post-balloon injury *in vivo*. (scale bar = 100 μm) (G) Intima transection area and (H) neointimal/media (N/M) ratio after arteries were balloon-injured. *p < 0.05; **p < 0.01: vs. sham group; ^#^*p <* 0.05; ^##^*p <* 0.01: vs. injured-3d or injured-14d group. All values are expressed as the mean ± SD, *n* = 5.

We further determined whether curcumin could inhibit neointimal hyperplasia and modulate miR-22 *in vivo*. Furthermore, we showed that miR-22 levels were significantly decreased at day 3 post-injury (Figure 6(A)), and SP1 levels were increased on day 3 in response to vessel injury (Figure 6(B,C)), while curcumin treatment reversed both those effects (Figure 6(A,C)). mRNA levels of α-SMA and calponin were reduced at 3 days following the injury, and such reductions were also rescued by curcumin (Figure 6(D,E)). Neointima formation was examined by H&E staining at 14 days after the balloon injury. Importantly, at 14 days post-balloon injury, we found that curcumin treatment significantly reduced the neointimal area and neointimal/media ratio compared with the injured group (Figure 6(F–H)). Taken together, these results indicate that the anti-neointima formation effects of curcumin are mediated by miR-22/SP1in response to vessel injury.

## Discussion

In this study, we revealed that the levels of miR-22 decreased in proliferative VSMCs and in the vascular wall following injury. We demonstrated that treatment with a miR-22 mimic could partially inhibit VSMC functions such as proliferation and migration through targeting SP1 *in vitro*. Moreover, we showed that curcumin treatment increased miR-22 levels and reduced SP1 expression in VSMCs. miR-22 inhibitors reversed the inhibition of VSMC proliferation and migration by curcumin. We finally demonstrated that the miR-22/SP1 pathway is involved, at least in part, in the protective role of curcumin during vascular injury *in vivo*.

It is well known that miR-22 is ubiquitously expressed in a range of tissues (Neely et al. 2006). It was then shown that miR-22 participates in angiogenesis, age-associated vascular diseases, and cardiac hypertrophy (Gurha et al. 2012; Zheng and Xu 2014; Takeda et al. 2016). Nevertheless, whether miR-22 plays a role in the dedifferentiation of VSMCs was not established. Here, we confirmed that miR-22 could inhibit VSMC proliferation and migration. We further observed that the expression of miR-22 correlates with the expression of α-SMA and calponin. Upon vascular injury, SP1 binds to G/C-rich repressor elements and decreases SM gene expression (Wamhoff et al. 2004). Deaton et al. demonstrated that SP1 binding to the Kruppel-like factor 4 (KLF4) promoter increases in platelet-derived growth factor (PDGF)-BB-stimulated SMCs following aortic balloon injury *in vivo* (Deaton et al. 2009). SP1 encodes PU.1, a transcriptional factor of the ETS family, which can control the transcription of specific genes by itself or by interacting with other transcriptional factors such as NF-κB or C/EBPα (Rothenberg et al. 2019). Our data confirmed that miR-22 directly targets SP1 and suppresses the proliferation and migration of VSMCs. These results indicate the miR-22/SP1 axis functions as a novel regulatory signalling pathway during VSMC proliferation and migration. Recently, Yang et al. (2018) also reported similar data in which miR-22 was found to regulate the dedifferentiation of VSMCs and the formation of vascular neointima in mouse models. Huang et al. (2017) found that the expression of miR-22-3p decreases in arteriosclerosis obliterans arteries, indicating that miR-22-3p can regulate the proliferation, migration, and neointima formation by targeting HMGB1 in HASMC. The present study also provides evidence that curcumin can inhibit the proliferation and migration of VSMCs through miR-22.

Curcumin is a widely consumed polyphenol that has beneficial effects in cardiac disease by suppressing VSMC proliferation and neointima formation, and attenuating inflammation and oxidative stress (Jeong et al. 2012; Liu et al. 2012; Sheu et al. 2013; Yang et al. 2013). Curcumin has been shown to regulate the expression of multiple miRNAs in a variety of neoplastic and non-neoplastic diseases. Curcumin also regulates miRNA levels in pancreatic cells, increasing the expression of miRNA-22 and decreasing the expression of miRNA-199a (Sun et al. 2008). Moreover, curcumin exerts its effects on the miR-590-3p/CD40 axis to play a protective role in injured endothelial cells (Wu et al. 2017). In addition, Sheu et al. (2013) have shown that demethoxycurcumin can downregulate the FAK/PI3K/AKT axis and suppress VSMC migration and vascular neointima formation. Nevertheless, the specific mechanisms of curcumin on VSMCs remain unclear. We confirmed that curcumin inhibits neointimal hyperplasia and demonstrates its ability to up-regulate miR-22 and downregulate SP1 in the injured vascular wall and VSMCs. In addition, the inhibition of miR-22 attenuated curcumin-induced inhibition on VSMC function. Thus, we revealed that curcumin inhibited the proliferation and migration of VSMCs and vascular neointimal hyperplasia partially through miR-22/SP1 signalling. We also demonstrated the involvement of miR-22 in VSMC function in response to vascular injury *in vivo*. The exact molecular mechanism that underlies the regulation of miR-22 expression remains unclear.

Nevertheless, a potential limitation should be noted. Currently, there is no uniform reference for curcumin concentration or dose for *in vitro* and *in vivo* experiments. In a study by Liu et al. (2014), the rats were pre-treated with low dose (50 mg/kg, intraperitoneal injection) and high dose (100 mg/kg, intraperitoneal injection) of curcumin for 5 days. In the study by Tamaddonfard et al. (2012), intraperitoneal injections of curcumin at 100 and 200 mg/kg were used. Gaedeke et al. (2005) used doses of 10 to 200 mg/kg body weight by intraperitoneal injection from days 3–5 after induction of disease, and the maximal inhibition doses were 50–100 mg/kg. Ray Hamidie et al. (2015) treated their rats with curcumin at 50 or 100 mg/kg-BW/day. Yu et al. (2011) used a dose of 50 mg/kg/day. He et al. (2010) used 50 mg/kg/day in 2-day-old neonatal Sprague-Dawley rats subjected to intracerebral injection of LPS. Samini et al. (2013) used an i.p. bolus of curcumin 50 or 100 mg/kg/day. Therefore, the present study selected a dose of 50 mg/kg/day.

## Conclusion

We demonstrated that miR-22/SP1 is a novel regulatory pathway during the proliferation and migration of VSMCs *in vitro*. Moreover, curcumin was found to increase the level of miR-22 and inhibit VSMC function and neointima formation *in vivo*. This study demonstrated the potential of curcumin in clinical interventions for vascular diseases, particularly during restenosis after coronary intervention.

## Supplementary Material

Figure_S2.tif

Figure_S1.tif

## Data Availability

The data that support the findings of this study are available from the corresponding author, YTL, upon reasonable request.
