# Supplementary figures and images for: Curcumin inhibits proliferation, migration and neointimal formation of vascular smooth muscle via activating miR-22
Source: Pharm Biol. 2020 Jul 6;58(1):610–9. doi: 10.1080/13880209.2020.1781904 (PMC8641690; doi:10.1080/13880209.2020.1781904)

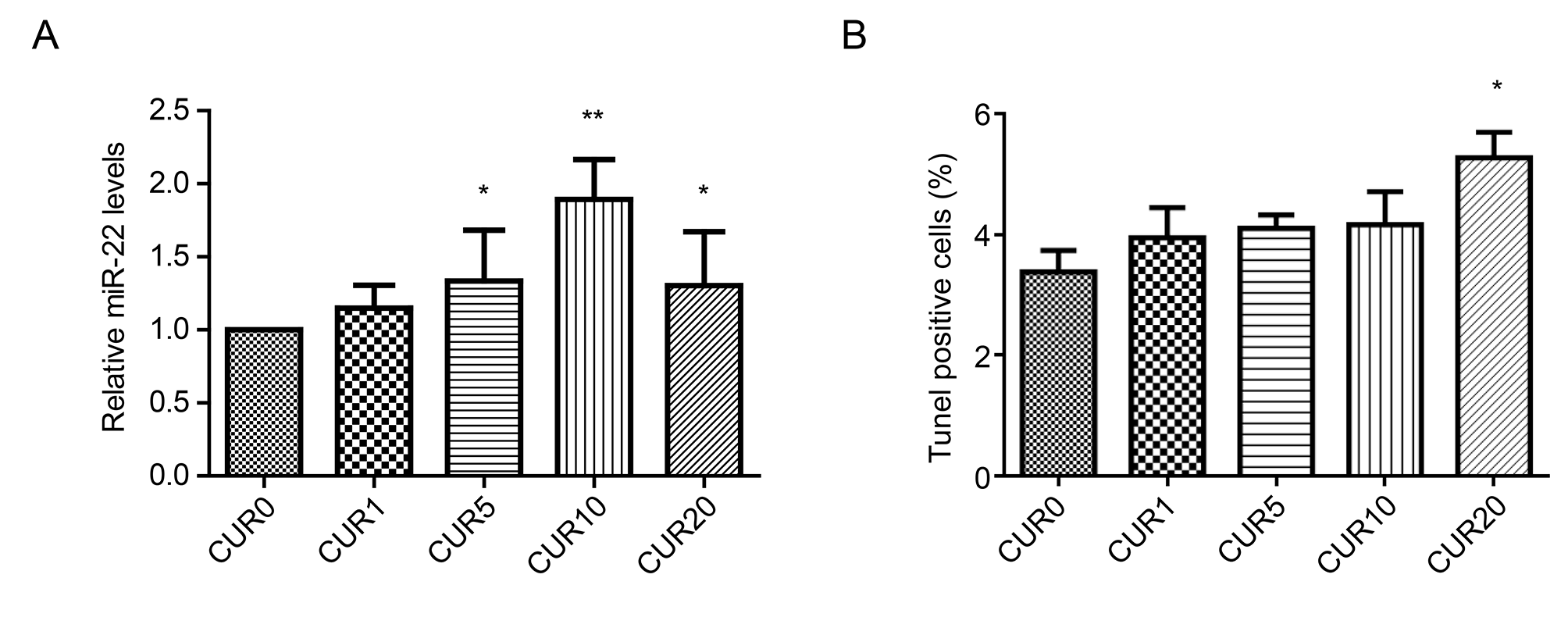

Supplement: Figure_S2.tif [file IPHB_A_1781904_SM0817.tif]

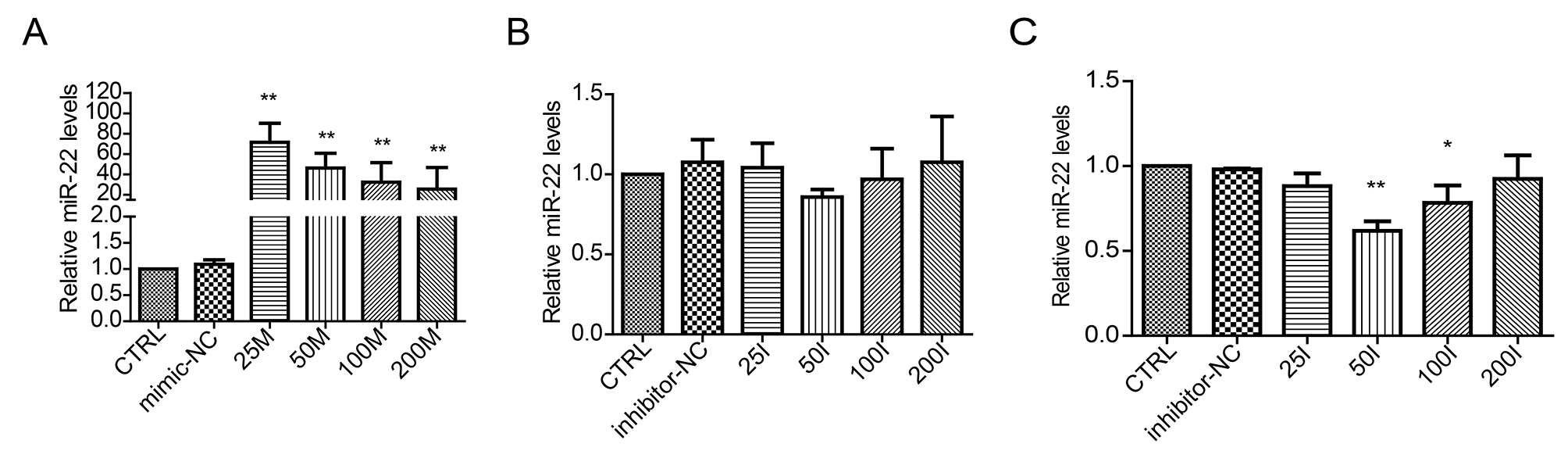

Supplement: Figure_S1.tif [file IPHB_A_1781904_SM0816.tif]
